# The Role of Biosimilars in Uveitis: Long-Term Real-World Outcomes of the Switch From Original to Biosimilar TNF-Alpha Inhibitors

**DOI:** 10.3389/fphar.2019.01468

**Published:** 2019-12-09

**Authors:** Claudia Fabiani, Antonio Vitale, Giacomo Emmi, Arianna Sgheri, Giuseppe Lopalco, Jurgen Sota, Silvana Guerriero, Florenzo Iannone, Bruno Frediani, Lorenzo Vannozzi, Maria Teresa Bianco, Valtere Giovannini, Gian Marco Tosi, Luca Cantarini

**Affiliations:** ^1^Ophthalmology Unit, Department of Medicine, Surgery and Neuroscience, University of Siena, Siena, Italy; ^2^Rheumatology Unit, Department of Medical Sciences, Surgery and Neurosciences, University of Siena, Siena, Italy; ^3^Department of Experimental and Clinical Medicine, University of Florence, Florence, Italy; ^4^Rheumatology Unit, Department of Emergency and Organ Transplantation (DETO), University of Bari, Bari, Italy; ^5^Department of Ophthalmology and Otolaryngology, University of Bari, Bari, Italy; ^6^Department of Surgery and Translational Medicine, Eye Clinic, University of Florence, Florence, Italy; ^7^Pharmacy Unit, Siena University Hospital “Santa Maria alle Scotte”, Siena, Italy; ^8^Azienda Ospedaliera Universitaria Senese, Le Scotte Hospital, Siena, Italy

**Keywords:** biosimilar, benepali, flixabi, imraldi, inflectra, originator, switch

## Abstract

**Background:** Recent expiry of patents for tumor necrosis factor (TNF)-α inhibitors has led to the employment of biosimilars in clinical practice. The aim of the study was to identify any change in the control of ocular inflammatory manifestations among patients with non-infectious uveitis switching from an originator to a corresponding anti-TNF-α biosimilar.

**Methods:** Thirty-seven consecutive patients (62 eyes involved) with non-infectious uveitis undergoing the switch from anti-TNF-α originators to biosimilars were retrospectively enrolled; the frequency of ocular flares before and after the switch as well as best corrected visual acuity (BCVA), central macular thickness (CMT), daily systemic corticosteroid intake, and frequency of uveitic macular edema (UME) at the switch and at the following assessments were statistically analysed.

**Results:** The number of ocular flares during the 12 months preceding the switch was 16, corresponding to 3.6 flares/100 patients/12 months; the number of flares after the switch was 14, corresponding to 2.0 flares/100 patients/12 months. No statistically significant differences were identified in the frequency of flares (*p* = 0.84) and in the number of patients experiencing ocular flares (*p* = 0.39) between the twelve months preceding the switch and the period thereafter. No statistically significant changes were observed in the BCVA (*p* = 0.27), CMT (*p* = 0.50), frequency of UME (*p* = 0.57) and daily corticosteroid intake (*p* = 0.42) between the time of the switch and the last follow-up visit.

**Conclusions:** The switch to biosimilars represents a feasible treatment choice associated with the maintenance of clinical efficacy in patients with non-infectious uveitis previously treated with the corresponding originator anti-TNF-α biologic agents.

## Introduction

Anti-tumor necrosis factor (TNF)-α biologic agents have shown to represent a primary option for the treatment of patients with non-infectious uveitis, especially in refractory cases (Fabiani et al., 2017; Bitossi et al., 2019; Fabiani et al., 2019a; Fabiani et al., 2019b; Fabiani et al., 2019c; Tosi et al., 2019). In particular, the monoclonal anti-TNF-α antibodies adalimumab (ADA) and infliximab (IFX) have been widely used to control intraocular inflammation, prevent ocular structural complications, avoid the impairment of visual function and allow a rapid tapering of systemic corticosteroids (Levy-Clarke et al., 2014; Dick et al., 2018).

Recent expiry of patents for anti-TNF-α originator biologic agents has led to development of similar versions of the original biopharmaceutical products, termed biosimilars. In particular, CT-P13 has been the first biosimilar monoclonal antibody to enter the market with the trade names of Inflectra^®^ or Remsima^®^. Later, additional biosimilar agents have been registered, including Flixabi^®^ (SB2, another biosimilar infliximab), Benepali^®^ (SB4, biosimilar etanercept) and Imraldi^®^ (SB5, biosimilar adalimumab). Physicochemical, pre-clinical, and clinical studies have proved similar efficacy, safety and immunogenicity between originators and biosimilars, thus leading to the approval of biosimilars for all indications of the reference product (Park et al., 2013; Yoo et al., 2013; Cho et al., 2016; Hong et al., 2017; Lee et al., 2019).

Beyond the significant costs associated with the use of originators, the development of biosimilar products has recently accounted for considerable cost savings and a substantial favorable impact on the healthcare budget; from this perspective, the number of patients possibly benefiting from an early access to biological treatment could be increased (Aladul et al.,2019). Nevertheless, introducing biosimilar agents to naïve patients and switching from the originator to a biosimilar gives rise to different medical liability issues. Indeed, switching from an option where the individual response is known to an option where it is unknown may expose patients to unpredictable risks, including loss of efficacy (Rocco et al., 2018). To date, studies addressing the consequences of the switch from originators to biosimilars have included patients with rheumatoid or psoriatic arthritis, spondyloarthritis and inflammatory bowel diseases (Emery et al., 2017; Jørgensen et al., 2017; Smolen et al., 2018; Weinblatt et al., 2018). Conversely, data related to patients with non-infectious uveitis are limited and the issue about ocular disease control after the switch is currently unexplored. In this context, the present study aimed at shedding light on this matter.

## Material and Methods

Patients suffering from non-infectious uveitis consecutively undergoing the switch from anti-TNF-α originator to biosimilar biologic agents were retrospectively enrolled; their demographic, clinical and therapeutic data were collected by accessing patients’ medical records.

The aim of the study was to identify any change in the control of ocular inflammatory manifestations in patients with non-infectious uveitis after the switch from an originator anti-TNF-α biologic agent to a corresponding biosimilar. The endpoints of the study corresponded to the identification of any: i) statistically significant difference in the frequency of ocular flares during the follow-up after the switch compared to the preceding 12 months by standardizing the rate of uveitis relapses as number of flares/100 patients/12 months; ii) statistically significant changes in the best corrected visual acuity (BCVA) and systemic corticosteroid intake (mg/day of prednisone or equivalent) between the time of the switch and the last follow-up visit; iii) statistically significant changes in the mean central macular thickness (CMT) and in the frequency of eyes with uveitic macular edema (UME) or retinal vasculitis at the last assessment (3-, 6- and 12-month follow-up visits) compared with the time of the switch.

The BCVA was expressed in decimal fractions by Snellen charts; CMT was assessed at optical coherence tomography performed at each follow-up visit; UME was diagnosed when CMT was >300 μm and presence of intraretinal fluid was confirmed after regular evaluation of the macular area. Active retinal vasculitis was diagnosed in case of evidence of retinal vascular leakage at fluorescein angiography. The anatomical and clinical classification of uveitis was established according with the standardization of uveitis nomenclature criteria (Jabs et al., 2005). Ocular flare was meant as an acute ocular inflammatory exacerbation occurred after a period of remission.

Descriptive statistics included sample size, percentages, means, and standard deviations. Normality distribution of quantitative data was assessed by using Shapiro-Wilk test followed by pair wise comparisons with unpaired two-tailed *t* test or Mann-Whitney two tailed U test, as appropriate. For categorical variables, comparisons were performed with Fisher exact test for 2 × 2 or 2 × 3 contingency tables. Two tailed *p*-values <0.05 were considered statistically significant.

The study has been approved by the local Ethics Committee of Azienda Ospedaliera Universitaria Senese, Siena, Italy (Ref. N. 14951). The study protocol was conformed to the tenets of the Declaration of Helsinki; informed consent was obtained from all patients enrolled.

## Results

Thirty-seven patients (21 males; 16 females) with a history of uveitis (62 eyes involved) were switched from originator anti-TNF-α biological agents to biosimilars as follows: Imraldi^®^ (n = 20 patients, 33 eyes involved); Flixabi^®^ (n = 10 patients, 16 eyes involved); Inflectra^®^ (n = 5 patients, 9 eyes involved); Benepali^®^ (n = 2 patients, 4 eyes involved). The follow-up duration of patients after the switch to biosimilars was 3 months in 20 patients, 6 months in 6 patients, 12 months in 11 patients. **Table 1** summarizes further demographic, clinical and therapeutic features of patients enrolled.

**Table 1:** Demographic, clinical and therapeutic features of patients enrolled.

Patients’ features	Years, mean ± SD
Age at the switch	43.8 ± 11.9
Age at uveitis onset	31.9 ± 12.7
Ocular disease duration	11.4 ± 7.1
Age at systemic disease onset	13.8 ± 12.4
Systemic disease duration	11.7 ± 8.7
Anatomical ocular involvement	Number of eyes involved, (%)
Anterior Uveitis	18 (29)
Intermediate Uveitis	2 (3.2)
Posterior Uveitis	23 (37.1)
Panuveitis	19 (30.6)
History of retinal vasculitis	16 (25.8)
Systemic disease’s features	Number of patients, (%)
Systemic disease related to uveitis	32 (86.5)
Behçet**’**s disease	26 (70.3)
Spondiloarthritis	5 (13.5)
Juvenile idiopathic arthritis	1 (2.7)
Idiopathic uveitis	5 (13.5)
Information about medications at the time of the switch	
Duration of treatment with originators, months (mean ± SD)	52.5 ± 38.9
Concomitant cDMARDs, n (%)	10 (27)
Methotrexate, n (%)	4 (10.8)
Cyclosporine A, n (%)	4 (10.8)
Azathioprine, n (%)	2 (5.4)
Corticosteroids (prednisone or equivalent), mg/day (mean ± SD)	4.1 ± 1.9

cDMARDs, conventional disease modifying anti-rheumatic drugs; SD, standard deviation.

Ocular flares involved 9 patients (5 treated with Remicade^®^, 3 with Humira^®^ and 1 with Enbrel^®^) during the 12 months preceding the switch to biosimilars and 6 (all treated with Flixabi^®^) patients thereafter. The number of ocular flares during the 12 months preceding the switch was 16, corresponding to 3.6 flares/100 patients/12 months; the number of flares after the switch to biosimilars was 14, corresponding to 2.0 flares/100 patients/12 months. No statistically significant differences were identified in the frequency of ocular flares (*p* = 0.84) and in the number of patients experiencing ocular flares (*p* = 0.39) between the twelve months preceding the switch and the period thereafter.

Ocular flares occurred while on Flixabi^®^ administration in all cases. None of the ocular flares developed within 3-month assessment; 12/14 (85.7%) ocular flares developed between 3-month and 6-month assessments; 2/14 (14.3%) ocular flares were observed between 6-month and 12-month follow-up visits. **Table 2** describes clinical features of eyes with uveitis at the time of the switch distinguished according with the biosimilar employed.

**Table 2:** Characteristics of eye involvement distinguished by different biosimilars employed at the time of the switch.

Biosimilars employed	Non-Anterior Uveitis, n (%)	Patients with BD, n (%)	Patients with idiopathic uveitis, n (%)	BCVA, mean ± SD	CMT, mean ± SD	UME, n (%)	Retinal vasculitis, n (%)	Eyes with uveitic complications, n (%)	cDMARDs coadministered, n (%)	Eyes with flares during the 12 months before, n (%)
Flixabi^®^ (n = 16)	12 (75)	13 (81.3)	3 (18.8)	7.23 ± 2.4	288.6 ± 35.6	5 (31.3)	6 (37.5)	4 (25)	5 (31.3)	3 (18.8)
Inflectra^®^ (n = 9)	9 (100)	9 (100)	0 (0)	6.9 ± 4.2	295 ± 30.3	2 (22.2)	0 (0)	3 (33.3)	2 (22.2)	1 (11.1)
Imraldi^®^ (n = 33)	22 (66.7)	22 (66.7)	4 (12.1)	9.3 ± 1.9	275.9 ± 43.1	2 (6.1)	0 (0)	5 (15.2)	7 (21.2)	7 (21.2)
Benepali^®^ (n = 4)	1 (25)	0 (0)	0 (0)	6.5 ± 2.1	287.5 ± 24.7	0 (0)	0 (0)	0 (0)	2 (50)	0 (0)

BCVA, best corrected visual acuity; BD, Behçet’s disease; cDMARDs, conventional disease modifying anti-rheumatic drugs; CMT, central macular thickness; n, number of eyes; UME, uveitic macular edema.

**Figure 1:**
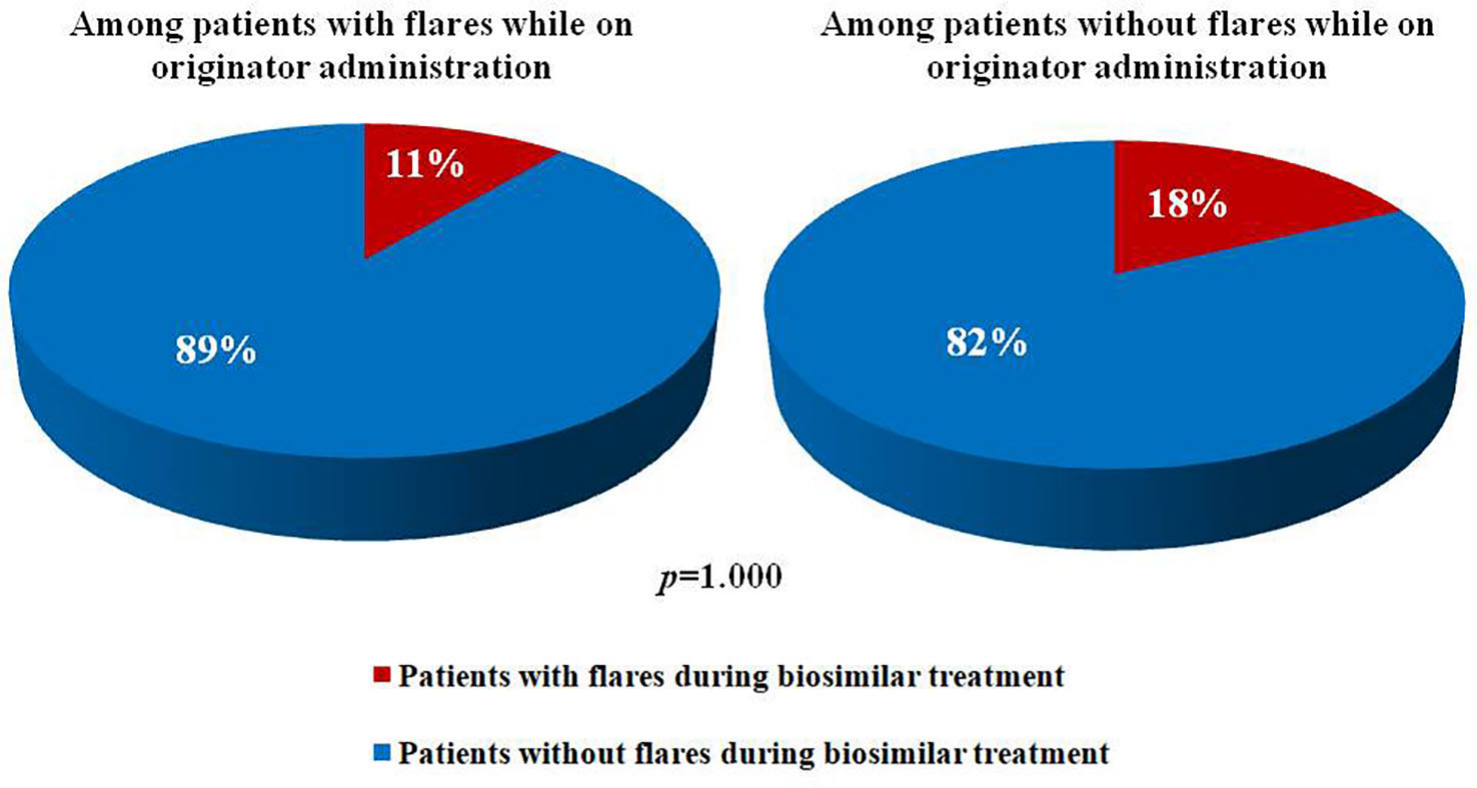
Pie charts illustrates percentages of patients experiencing and not experiencing ocular flares after the switch from an originator anti-tumor necros factor-α to a corresponding biosimilar among patients with and without uveitic flares during the 12 months before.

**Figure 1** illustrates percentage of subjects experiencing ocular flares after the switch among patients with and without flares during the previous 12 months. No statistically significant differences were observed between the two groups (*p* = 1.000). Similarly, no statistically significant differences were observed in the frequency of flares preceding the switch between patients with and without flares afterward (17% and 26%, respectively, *p* = 1.000).

The mean BCVA was 8.4 ± 2.5 decimals at the time of the switch and 8.5 ± 2.48 decimals at the last assessment (*p* = 0.27).

Retinal vasculitis was found in 3 patients (6 eyes, all treated with infliximab) at baseline; 4/6 eyes had an associated UME. All of these patients received 1 mg/kg/day of oral prednisone at the time of the switch. In all cases oral prednisone was gradually tapered to ≤5 mg/day within the first 3 months of follow-up. At the end of the study all of these patients had a follow-up equal or superior to 12 months with no relapses of retinal vasculitis at 3-, 6-, 12-month and last follow-up assessments.

The mean CMT was 281.4 ± 39.4 µm at baseline, 282.9 ± 31.6 µm at 3-month assessment, 275.5 ± 24.3 µm at 6-month evaluation, 275.9 ± 27.5 µm at 12-month follow-up visit (*p* = 0.50); UME was observed in 5 patients (9 eyes) at baseline and in one patient (2 eyes) at 3-, 6-, and 12-month assessments (*p* = 0.57).

**Table 3** provides information about BCVA and CMT as well as the frequency of UME and ocular relapses according to the different follow-up duration of patients enrolled.

**Table 3:** Best corrected visual acuity (BCVA), central macular thickness (CMT), frequency of uveitic macular edema (UME) and number of ocular relapses recorded between the time of the switch and the last follow-up assessment distinguishing patients according to the different follow-up duration.

Follow-up duration	BCVA at the switch	BCVA at last assessment	p-value	CMT at the switch	CMT at last assessment	p-value	UME at the switch	UME at last assessment	p-value	Number of ocular flares after the switch
3 months (n = 20)	10 ± 1.95	9 ± 2.1	0.109	275.9 ± 43.1	283 ± 37.7	0.859	2	0	0.468	0
6 months (n = 6)	6.6 ± 3.5	6.8 ± 3.4	0.317	280 ± 15	278.7 ± 14.5	0.414	1	0	> 0.99	0
12 months (n = 11)	7.3 ± 2.5	8.4 ± 2.7	0.014	294.4 ± 37.4	273.9 ± 28.5	0.056	6	2	0.184	14

“n” refers to the number of patients.

The mean daily corticosteroid dosage was 4.1 ± 1.9 mg/day at the time of the switch and 6.3 ± 7.8 mg/day at the last follow-up visit (*p* = 0.42). Nine patients took corticosteroids at the start of biosimilar agents and 8 patients at the last assessment (*p* = 0.78). At the time of the switch 10 patients (27.03%) were concomitantly treated with cDMARDs; at the last assessment, patients administered with cDMARDs were 9 (24.3%) (*p* = 0.79). Regarding the posology changes, an increase in the frequency of administration was required in 1 patient undergoing Inflectra^®^ treatment; conversely, 3 patients (2 treated with Flixabi^®^ and 1 treated with Inflectra^®^) decreased the frequency of administration and 1 patient treated with Flixabi^®^ underwent a decrease of the dosage from 5 mg/kg/8 weeks to 3 mg/kg/8 weeks. Conversely, no posology changes had been performed during the 6 months preceding the switch.

At the time of the switch, ocular structural complications as consequence of uveal inflammation were present in 9 patients (epiretinal membranes in 7 eyes; macular hole in 2 eyes; retinal atrophy in 2 eyes; retinal atrophy and detachment of retinal pigment epithelium in 1 eye). In addition, age-related macular degeneration had been observed in 1 eye. At the last assessment new uveitic ocular complications arose in 2 patients (3 eyes) treated with Imraldi^®^. In particular, epiretinal membranes were observed in 2 eyes and lens opacity in 1 eye.

No severe adverse events were observed following the switch to biosimilar agents.

## Discussion

The issue about clinical consequences of switching from originators to biosimilar biologic agents currently represents a central topic for scientific debate. However, this topic has mainly been concentrated on patients with rheumatoid or psoriatic arthritis, spondyloarthritis, and inflammatory bowel diseases. On the contrary, data about the switch in patients with non-infectious uveitis are quite scarce in spite of the increasing importance of the anti-TNF-α agents adalimumab and infliximab for the treatment of non-infectious uveitis (Fabiani et al., 2018).

Our results suggest an optimal maintenance of uveitis control following the switch from originators to biosimilars. In particular, the rate of uveitic flares observed during the 12 months preceding the switch and the subsequent follow-up period were comparable (3.6 and 2.0 flares/100 patients/12 months, respectively) with no statistically significant differences. Similarly, visual acuity was maintained at the last follow-up visit compared to the time of the switch. Furthermore, the CMT values and the frequency of UME did not change significantly after the switch. Likewise, the mean daily corticosteroid intake did not change significantly, thus ensuring the persistence of the corticosteroid sparing effect during treatment with anti-TNF-α biosimilars.

Retinal vasculitis was identified bilaterally in 3 patients successfully treated with short-term oral corticosteroids at the time of the switch. Of note, no relapses of retinal vasculitis were identified thereafter (the follow-up while on biosimilar treatment lasted at least 12 months in all three cases).

As a whole, our results confirm previous findings obtained from a prospective observational study conducted in 8 patients with uveitis followed for a mean period of 2.9 years (Avouac et al., 2018). In particular, according to Avouac et al. (2018), no statistically significant changes were observed in the uveitis activity score and in the number of ocular flares between the time of the switch and the last visit. In the same way, basing on data from 13 patients with Behçet’s disease (10 of them with uveitis), Lopalco et al. (2018) found that switching from reference infliximab to biosimilar CTP-13 was a feasible and uneventful possibility, with no ocular relapses and no significant changes of disease activity during a nine-month follow-up period.

Of note, in our study the frequency of patients with ocular flares after the switch was similar among patients with and without flares during the 12 months preceding the switch. In the same way, the frequency of ocular flares prior to the switch was comparable between patients with and without flares thereafter. As a consequence, the probability of experiencing ocular flares after the switch appears independent of disease control prior to the switch. These findings do not contradict the possibility of ocular flares in patients experiencing full therapeutic success with originators, as previously reported by Cantini et al. (2017).

Noteworthy, in the present study all of the ocular flares recorded after the switch were observed while on the Flixabi^®^ administration. Further studies specifically addressing this issue will have to clarify whether this finding is a result of chance. However, the worst performance obtained with Flixabi^®^ could be related to the worst-case ocular conditions recorded at the time of the switch among patients undergoing this biosimilar infliximab. Indeed, as observed in **Table 2**, the frequency of retinal vasculitis, UME, Behçet’s disease diagnosis and non-anterior uveitis supports a more severe baseline clinical context among patients switched to Flixabi^®^ (Tugal-Tutkun, 2009; Dick et al., 2016; Fardeau et al., 2016; Dick et al., 2018).

The main limitation of the study is represented by its retrospective design. In addition, our study did not include an evaluation of multiple therapy switches between the biosimilar and the originator product or between different biosimilars. Nevertheless, to the best of our knowledge this is the first study primarily addressing the impact of switching from originators to biosimilars in terms of frequency of ocular flares, visual acuity, CMT, daily corticosteroid dosage and occurrence of UME in a fair number of patients with non-infectious uveitis.

In conclusion, the switch to biosimilars represents a feasible treatment choice associated with the maintenance of clinical efficacy in patients with non-infectious uveitis previously treated with the corresponding originator anti-TNF-α biologic agents.

## Data Availability Statement

The datasets generated for this study are available on request to the corresponding author.

## Ethics Statement

The studies involving human participants were reviewed and approved by Azienda Ospedaliera Universitaria Senese. The patients/participants provided their written informed consent to participate in this study.

## Author Contributions

CF and AV wrote the manuscript. LC and CF designed the study. CF, LC, MTB, and VG finally revised the manuscript. AV performed the data analysis. CF, LC, AV, GMT, JS, SG, GL, AS, GE, FI, BF, and LV took care of patients’ enrollment, follow-up of the patients, and data collection.

## Conflict of Interest

The authors declare that the research was conducted in the absence of any commercial or financial relationships that could be construed as a potential conflict of interest.
